# The Success Rate and Factors Affecting the Outcome of Assisted Reproductive Treatment in Subfertile Men

**Published:** 2020-02

**Authors:** Alireza ZARINARA, Hojjat ZERAATI, Koorosh KAMALI, Kazem MOHAMMAD, Maryam RAHMATI, Mohammad Mahdi AKHONDI

**Affiliations:** 1. Reproductive Biotechnology Research Center, Avicenna Research Institute, Tehran, Iran; 2. Department of Epidemiology and Biostatistics, School of Public Health, Tehran University of Medical Sciences, Tehran, Iran; 3. Department of Public Health, School of Public Health, Zanjan University of Medical Sciences, Zanjan, Iran; 4. Department of Epidemiology and Biostatistics, School of Public Health, Tehran University of Medical Sciences, Tehran, Iran

**Keywords:** Reproduction, Infertility treatment, Male subfertility, Prognostic factors

## Abstract

**Background:**

This study was conducted to evaluate the success rate of male infertility treatment and the factors affecting its outcome.

**Methods:**

In a historical cohort study, from Mar 2013 to Mar 2014, 323 couples with male factor were investigated. Couples had treated with IUI or/and ICSI were included randomly. Assisted reproduction technology (ART) outcome (treatment success) was defined as a live birth. Age, duration of infertility, type of infertility, treatment history and clinical examination results were investigated. The logistic regression and survival analysis were applied.

**Results:**

The average of men age, duration of infertility and BMI were 33.5, 4.7 (yr) and 26.6 (kg/m^2^) respectively. 87.9% of men have primary infertility and average duration of treatment was 14.1(month). Previous treatment, type of infertility, treatment method, man’s BMI, normality of sperm and sperm head were important variable that affecting outcome. The rate of live birth in the first attempt was 29.7%, and 44.9% of the couples succeeded to give live birth after several treatment cycles. Couples who had no previous history of treatment were 8.5 times more successful in live birth. The Cox analysis showed that “BMI of man” and percentage of “Sperm with normal head” are predictors that had a significant effect on live birth.

**Conclusion:**

Live birth in the first treatment cycles was influenced by four variables but two other variable were affecting several treatment cycles outcome. The chances of successful treatment were higher with taking into account the length of time and having live birth was determined as 78% for five years of continuous treatment.

## Introduction

Male infertility has numerous causes and these causes can change the type of treatment needed and its success rate (1, 2). Several Assisted Reproductive Technologies (ARTs) are available today that help eliminate the effect of the factors causing infertility from the reproductive cycle.

The success rate of ART increases with the correct diagnosis of the cause of infertility. In some cases, however, the cause may not be identified or several factors may be concurrently involved to make the treatment complex, lengthy and difficult. Identifying the factors affecting the success of ART treatment success requires a proper understanding of the factors involved (3). More than 20% of couples in Iran have faced with subfertility (2). More than half of the causes of infertility are related to male factor (4); widespread causes of subfertility in women have led to more research and the main reason for men’s subfertility has been ambiguous. Consequently, male subfertility has been less studied independently and the success of ART treatment with male factor is a topic less addressed.

Studies conducted over the past three decades have tried to determine the chances of successful reproduction before beginning treatment or after it. In the case of the latter, researchers have often considered the success rate of a particular treatment, have compared the existing conditions and have examined the effect of external factors on that particular treatment or on a combination of treatments (5). The majority of these studies have used a simple ratio-based approach while some have measured probabilities for determining the chances of a successful pregnancy. Using the simple ratio-based approach for determining the likelihood of treatment success is justified if the aim is to compare the success rate of different methods or to compare them with each other(6); however, determining the likelihood of success based on the predictive factors helps use those factors for the analysis of the success rate. Male factors are studied based on semen specifications and sperm quality (7, 8). Age, occupational hazards and some other factors have an effect on outcome of ART treatment(9, 10). Consequently, identifying the factors affecting the success of ART treatment is highly important and determining the probability of treatment success, requires a proper understanding of the factors involved (11, 12).

Today, as technology progresses, the use of ART treatment is increasing. Two ART plans (IUI and ICSI) have more use for subfertility treatment with male factor. In many cases, these treatments are used one after the other (even sometimes with repetition). Therefore, the general success rate of treatments and the effective factors are crucial.

In Iran, infertility treatment methods in different centers have been widely practiced, but the rate of treatment success has not been officially reported. The success rate can vary from center to center and will change by definition of success and method of treatment. Moreover, treatment success can be changed with type and repetition of treatment. Not only in Iran, but elsewhere, we did not find the rate of success in treating male infertility in general.

Apart from semen specifications, it seems several factors can change the outcome of ART treatment and the aim of this study was to determine the general success rate of ART treatments (IUI and ICSI either singly or both) in order to investigate the factors affecting treatment success by use of the data obtained from Iranian population.

## Materials and Methods

In this historical cohort study, from Mar 2013 to Mar 2014 couples had treated with IUI or/and ICSI were included in the study. They met the criteria as follow:

### Inclusion criteria

- Male factor infertility
- Age 40 yr and less for woman
- A healthy reproductive system for woman
- Treated with homologous sperm, ovum and embryo
- Treated with good quality (fresh or frozen-thawed embryo)

### Exclusion criteria

- Previous ART treatments for woman (not healthy reproductive system)

In Jul 2015, the medical records of 323 couples with male factor infertility were randomly selected and reviewed. The records were selected in proportion to the number of patients admitted each month. Sample size was calculated with 5% error, 95% confidence interval and 30% probability of successful treatment.

Overall, 31 variables including the demographic data and other factors affecting pregnancy such as hormonal tests, type of infertility (primary and secondary in man), treatment history and physical examination were reviewed.

ART outcome or treatment success was defined as a nominal qualitative variable including clinical pregnancy and live birth requiring two criteria as noted:

- Clinical pregnancy: Fetal heart rate in the ultrasound by the seventh week of gestation
- Live birth: Giving birth to a live baby by vaginal delivery or caesarean section after 38 wk of pregnancy

### Statistical analysis

Quantitative data expressed as mean (standard deviation) and median (inter quartile range). All continuous variables were checked for normality using the one-sample Kolmogorov–Smirnov test. Due to the lack of normal distribution, data were analyzed by non-parametric test (Mann-Whitney) and univariate logistic regression and presented as mean±SD and median. Qualitative data were analyzed using Chi-square test. Multiple logistic regression was used for analyzing the simultaneous effects of variables on delivery. The survival analysis (Life table, Kaplan-Meier and Cox regression) was used to study the waiting time for successful pregnancy and the factors affecting it. The level of statistical significance was set at P<0.05 for all the tests. The data obtained were analyzed in SPSS ver.16 (Chicago, IL, USA)

This study was approved by Ethical Committee of Avicenna Research Institute (ARI). Specialists in Avicenna Fertility Center briefed the patients and obtained their informed consent for the recommended treatment. Treatment information was extracted from patients’ records with their consent.

## Results

Demographic information and some characteristics of the samples are listed in Table 1.

**Table 1:** Demographic data and some specifications of couples

	***Subject***	Mean (±SD) or number (%)/ Median (IQR)^*^
1.	Age of men Age of women	33.5(±5.6)(years)/ 33(7) 28.8(±4.5)(years)/ 29(7)
2.	Men’s BMI Women’s BMI	26.6(±3.9)(kg/m^2^)/ 26.9(3.5) 25.2(±3.9)(kg/m^2^)/ 24.6(5.6)
3.	Duration of marriage Duration of infertility	6.26(±3.9)(years)/ 5(5) 4.76(±3.9)(years)/ 3(3)
4.	Men’s birthplace Women’s birthplace	Tehran 29.7% (The rest 70.3%) Tehran 29.4% The rest 70.6%)
5.	Men with previous treatment at other medical centers Men with no history of infertility in close relatives Women with no history of infertility in close relatives	12 Case (3.7%) 278 Case (86.06%) 298 Case (92.3%)
6.	Men with interfamilial marriage (i.e. marriage with their uncle or aunt’s daughters)	54 Case (16.8%)
7.	Smoker men	44 Case (13.6%)
8.	Men with varicocele (Mild to moderate) Men with testis atrophy and orchidectomy	29 Case (8.97%) 27 case (8.37%)
9.	Men with history of surgery in pelvic area	Total 107 Case (33.1%) Varicocelectomy 97 Case (30%) Inguinal hernia 10 Case (3.1%)
10.	Differential diagnosis based on urologist report	Teratospermia (19.51%) Oligoasthenospermia (14.86%) Azoospermia (13.94%) Asthenoteratospermia (12.38) Asthenospermia (2.16%) Pyospermia (1.55%) Oligospermia (1.23%) No report (Undefined) (32.20%)
11.	Women with healthy reproductive system	323 (100%)(Main inclusion criteria)
12.	Men with secondary infertility	39 Case(12.1%)(Etiology was not specified)
13.	IUI as the first treatment ICSI as the first and only treatment	118 Couples (36.5%) 205 Couples (63.5%)
14.	Duration of treatment	14.1(±16.1) months (median 9.9months)
15.	Clinical pregnancy in the first attempt Delivery in the first attempt	119 Case 96 Case
16.	The success rate in the first attempt (clinical pregnancy) The success rate in the first attempt (live birth)	36.8 per Couple(per cycle) 29.7% per Couple(per cycle)
17.	Clinical pregnancy after several attempts	176(54.5%) (Sample may have been positive result twice or more)
18.	Delivery after several attempts	146 Case
19.	The success rate after several attempts (live birth) 631 treatment cycles were administered	45.2% per couple 23.1% per cycle

* Median(IQR) of successful couple

### Success rate

**Fig. 1:**
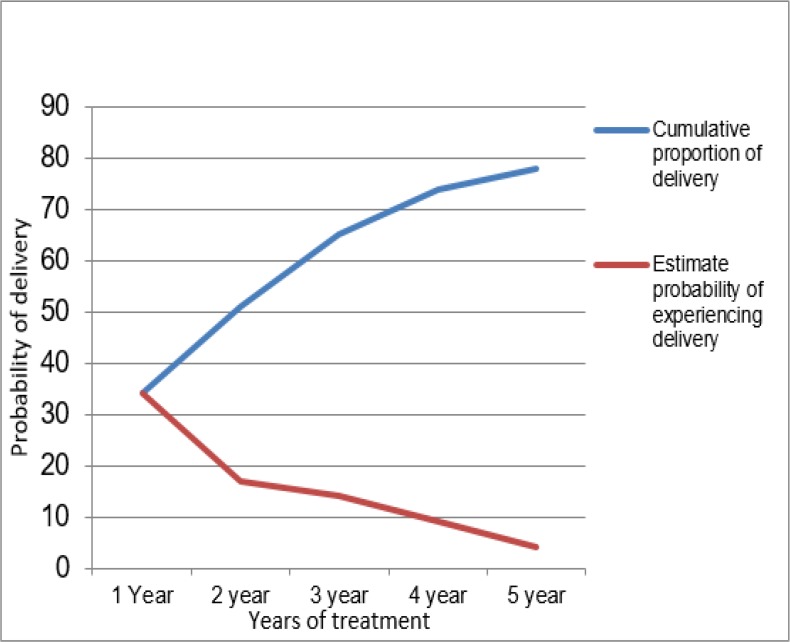
The cumulative rate (%) of cases experiencing delivery and probability of treatment success

The chance of successful treatment (i.e. live birth) and the factors affecting time to live birth were investigated using the survival analysis. The cumulative proportion of cases experiencing delivery from the start of the treatment to the end of the interval was 34% during the first year, 65% in the first three years, and 78% in the first 5 years (Fig. 1, blue line shows an increase in the cumulative proportion of live birth over time).

**Fig. 2:**
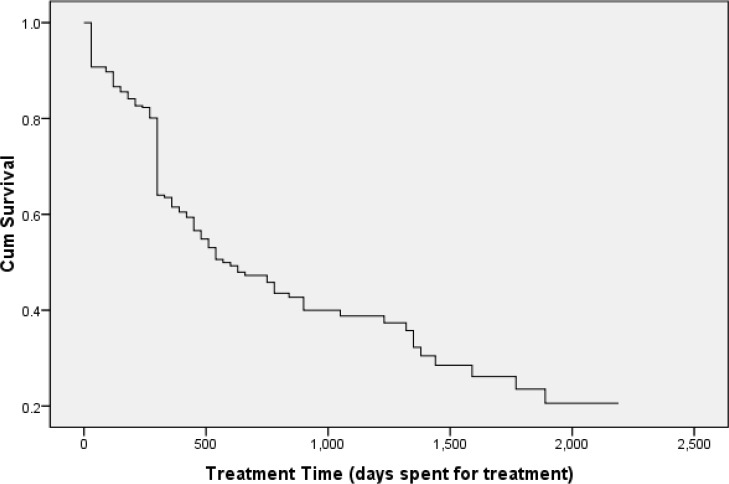
Relationship between the duration of treatment and the remaining couples for treatment process. When treatment time is increased, the likelihood of a couple staying in the treatment process is reduced

Moreover, year by year, this probability has decreased. Therefore, that it reached 0.17, 0.14, 0.09, and 0.04 during 2nd, 3rd, 4th, and 5th interval, respectively (Fig. 1: Over time, reducing the likelihood of live birth is shown with red line). As treatment time is increased, the likelihood of a couple staying in the treatment process is reduced. Fig. 2 shows the fraction of the patients that not experiencing delivery for a certain amount of time after treatment. By Kaplan-Meier method, the median treatment duration was 16±2.3 months (CI95%: (11.4,20.6)).

### Effective factors

Thirty-one variables expected to be effective in the success of treatment were analyzed. Fourteen variables that had a probability value less than 0.25 in Mann-Whitney or Chi-square tests were selected as the factors affecting the treatment success and analyzed (Table 2).

**Table 2:** Univariate logistic regression and Cox regression analysis with considering the clinical pregnancy and live birth as a success

	***Variable(Factor)***	***Univariate logistic regression (first attempt)***				***Univariate Cox-regression Live birth***			
		***Clinical pregnancy***		***Live birth***					
		***P-value***	***OR^a^***	***P-value***	***OR^a^***	***P-value***	***HR^b^***
1	Previous infertility treatment of man	0.040	3.604	0.037	3.492	0.081	0.562
2	Secondary infertility of man	0.021	2.214	0.046	2.005	0.182	0.773
3	IUI as the first ART treatment	0.003	2.111	0.012	1.971	0.875	1.029
4	No Familial marriage	0.121	1.596	0.205	1.486	0.723	0.929
5	FSH(mlu/ml)	0.142	0.968	0.221	0.972	0.487	0.986
6	LH(mlu/ml)	0.124	0.927	0.215	0.939	0.994	1.000
7	Man’s BMI(kg/m^2^)	0.205	1.041	0.018	1.083	0.057	1.045
8	Infertility duration of man	0.102	0.950	0.203	0.959	0.097	0.962
9	Normal form of sperm(%)	0.140	0.968	0.374	0.980	0.029	0.967
10	Sperm with normal head(%)	0.217	0.985	0.283	0.986	0.001	0.973
11	Sperm with good motility(%)	0.102	0.981	0.529	0.994	0.972	1.000
12	Live sperm(%)	0.026	0.976	0.286	0.989	0.586	1.001
13	Total testis volume	0.295	1.017	0.448	1.012	0.238	1.011
14	Duration of marriage	0.165	0.958	0.359	0.971	0.570	0.988

a: Odds ratio

b: Hazard rate

In univariate cox-analysis, two variables had a significant effect on live birth (yellow) and four variables had probability value of less than 0.25 (pink). Univariate logistic regression for variables showed five variables significantly affecting the treatment success (i.e. clinical pregnancy and live birth) in the first attempt (yellow). Eight variables, as marked (blue) in table 2 were evaluated in delivery (As the dependent variables) using multiple logistic regression (The backward Wald method). In terms of success as defined by clinical pregnancy, “The type of male infertility” (i.e. primary or secondary) and “The type of ART treatment” were significantly effective variables in the first attempt. In terms of success as defined by live birth, “The man’s BMI” and “The type of ART treatment” were the effective factors that were significant (Table 3).

**Table 3:** The multiple logistic regression of the selected variables in the couples’ first attempt

***Definition Success***		***Effective variable***	**P*-value***	***OR^a^***	***95%CI^b^ for OR***	
					***Lower***	***Upper***
Clinical pregnancy	1	Type of male infertility	0.006	3.605	1.373	6.841
	2	Type of ART treatment	0.028	1.868	1.069	3.267
Live birth	1	Type of male infertility	0.025	2.451	1.119	5.371
	2	Man’s BMI	0.008	1.104	1.026	1.188

a: Odds ratio

b: Confidence interval 95%

The chance of clinical pregnancy was 3.605 times higher in men with secondary infertility and 1.868 times higher in those who had undergone IUI. Men with secondary infertility had a higher chance of success in terms of live birth (Table 3). The chance of live birth increased 1.104 times per each unit of increase in the man’s BMI and increased 2.451 times higher in those with secondary infertility. Three factors appear to predict couples’ success in their first attempt, including “the type of male infertility”, “the type of treatment”, and “the man’s BMI”.

Multiple logistic regression analysis of the selected variables for live birth with considering all the treatment attempts showed “The man’s previous history of infertility treatment” is an effective factor and the probability of success in live birth was 8.5 times higher in couples who had no history of treatment (*P*=0.045, OR=8.5, CI95%: 1.046–69.053) (Not presented in table).

## Discussion

The success rate is calculated only for one treatment method, but in this study, we computed treatment success rates with a general approach to IUI or ICSI or both. Additionally, in our study, the effect of repeat of treatment (duration of treatment) is also considered. This approach has yielded more practical results. Multiple logistic regression and Cox-analysis was used for showing simultaneous effect of variables (13–15) In this study, eight variables were examined that other researchers had proposed for the successful treatment (16–18). These variables as effective factors can be used as predictors(19).

An attempt was made to control the maternal factors by the couple selection criteria. The age range and the healthy reproductive system of women show this control (Table 1). Men suspected of fertility (Healthy) were excluded.

When the exact definition for the outcome of the treatment is provided, the factors that affect the treatment become clearer. For this reason, two distinct criteria for the success of treatment were considered. Conception according to the ultrasound results as clinical success and baby delivery (Live birth) as the final outcome were included as well (8). The birth of the baby is greatly influenced by the events during pregnancy, the factors related to the mother’s body and fetal growth conditions (20). This can affect the success rate for live birth and depends on factors unrelated to male infertility (21, 22). The factors affecting clinical pregnancy results provide a more realistic predictive tool for couples and physicians (23).

Women’s increased age reduces fertility (24); however, this fact does not apply to men (25) but men’s fertility decreases with age (26). To control the effect of this factor, women’s age and the couples’ mean age were limited within the fertility age range.

Obesity is a consequence of some genetic diseases also associated with infertility and has an adverse effect on male fertility (27). Male obesity is emphasized by physicians in the process of infertility treatment so as to facilitate the success of the treatment (OR=1.104 in Table 3). The couple’s BMI, which is often negatively correlated with fertility outcomes (20, 28), was not a confounding variable in the present study, as the mean BMI was normal in half of the women while others were only mildly overweight. Obesity appears to have a greater adverse effect on fertility in men (28). In this study, only 5% of the men had a BMI above 33 and the increase in BMI increases the chances of success by 6%. Perhaps this interpretation is surprising at first glance, but 41% of men had BMI lower than normal level and 3% were obese. Thus the increase in BMI has brought men’s weight closer to normal for achieving success. This justification has also been raised in MacDonald et al (29) investigations. The interpretation needs to be more carefully considered as complementary research.

In our research, family marriages (marriage with aunt or uncle’s daughter) have not significant effect on treatment success rate. Although research has been emphasized on the fate of pregnancy in family marriages by factors may activate immunologic or ambiguous factors (30).

We know despite several attempts for treatment, couples who have not been successful in pregnancy have a smaller chance of success (17). The man’s previous history of infertility treatment was proposed as a predictor of treatment success (31), and the same result was obtained in our study.

Semen analysis, the examination of diseases affecting male infertility and the testicular pathological evaluation as a complementary criterion for judging the quality of sperm can help to find effective factors (32), although in our study variables related to these topics were not significant, further research is recommended.

The results showed that the factors affecting treatment success in several attempts are not similar to the factors determined for the first attempt; while McLernon has introduced similar variables (17). In our study, more variables were introduced when the results of several attempts were analyzed.

According to the survival analysis (Fig. 1, 2), the probability of success has been 78% in a five-year period and patient characteristics have no effect on treatment success. Moreover, couples’ characteristics determine the type of infertility and some of the treatment options available and putative factors cannot be manipulated or changed (8). In this analysis, treatment success was more than the ratio in the first treatment. As with another study, the success rate in a five-year period was more than the first attempt (33).

Although different variables may affect treatment success in the early years of treatment, the Cox analysis performed based on the duration of treatment showed that two variables, “BMI of man” and percentage of “Sperm with normal head” affect the duration of treatment and like other findings (34, 35). It seems these variables which have significant effect in this analysis are more important for consideration and application.

The chance of having live birth after ART treatment was determined as 78% for five years of continuous treatment. The variables that influenced the outcome of the first and several treatment cycles were different.

## Conclusion

The success rate of ART treatments is low regardless of the length of treatment. The success rate is greater with regard to the length of time and the repetition of treatment and is closer to reality.

## Ethical considerations

Ethical issues (Including plagiarism, informed consent, misconduct, data fabrication and/or falsification, double publication and/or submission, redundancy, etc.) have been completely observed by the authors.
